# Supplementary material for: Effect of Mobile Phone Addiction on Physical Exercise in University Students: Moderating Effect of Peer Relationships
Source: Int J Environ Res Public Health. 2023 Feb 2;20(3):2685. doi: 10.3390/ijerph20032685 (PMC9915063; doi:10.3390/ijerph20032685)
Supplement: Supplementary file 1 [file ijerph-20-02685-s001.zip › ijerph-2179584-supplementary.pdf]

Mobile Phone Addiction Tendency Scale (MPATS)

1 If I haven't brought my phone with me for a while, I will check for text messages or missed calls immediately.

2 I'd rather chat on my phone than face to face.

3 When waiting for someone, I often call my mobile phone to ask where the other person is, and if I don't call, I am in a hurry.

4 If I haven't used my phone for a long time, I feel bad.

5 In class, I can't concentrate because of my mobile phone.

6 I would feel lonely without a mobile phone.

7 I feel more confident when I communicate with others on my phone.

8 When WeChat doesn't ring for a while, I will feel uncomfortable and subconsciously look to see if there are missed calls or text messages.

9 I often have the illusion that my phone is ringing or my phone is vibrating.

10 More phone calls, more text messages, I will feel more fulfilling in life.

11 I am often afraid of my phone turning off automatically.

12 The phone is a part of me, and once I reduce it, I feel like I've lost something.

13 My classmates and friends often say that I am too dependent on my mobile phone.

14 When my phone is often not connected or has no signal, I become anxious and grumpy.

15 In class, I often actively focus on my mobile phone and affect my listening to the class.

16 I feel more comfortable communicating with others on my mobile phone.

PhysicalActivity Rating Scale (PARS-3)

1. How intense do you usually exercise physically?

○ Light exercise (e.g. walking, doing radio exercises, playing gateball, etc.)

○ Less intense sports of low intensity (recreational volleyball, table tennis, jogging, etc.)

○ Moderate intensity of intense sustained exercise (e.g. cycling, running, table tennis)

○ Shortness of breath and sweating a lot of high-intensity but not lasting sports (such as playing badminton, volleyball, etc.)

○ Shortness of breath, sweating a lot of high-intensity sustained exercises (such as running, calisthenics exercises, swimming, etc.)

2. How many minutes do you usually engage in the above intensity physical activity?

○ 10 minutes or less

○ 11 to 20 minutes

○ 21 minutes to 30 minutes

○ 31 minutes to 59 minutes

○ 60 minutes or more

3. How many times do you usually engage in the above physical activities?

○ Less than 1 time a month

○ 2-3 times a month

○ 1-2 times a week

○ 3-5 times a week

○ Approximately l times a day
